# A Role for Mesenchyme Dynamics in Mouse Lung Branching Morphogenesis

**DOI:** 10.1371/journal.pone.0041643

**Published:** 2012-07-23

**Authors:** Pierre Blanc, Karen Coste, Pierre Pouchin, Jean-Marc Azaïs, Loïc Blanchon, Denis Gallot, Vincent Sapin

**Affiliations:** 1 Retinoids, Development and Developmental Diseases – EA 7281, Medicine School, Auvergne University, Clermont-Ferrand, France; 2 Department of Biochemistry and Molecular Biology, University Hospital, Clermont-Ferrand, France; 3 GReD, CNRS 6247, INSERM 1103, UMR, Clermont University, Clermont-Ferrand, France; 4 Mathematic Institute, Toulouse III University, Toulouse, France; 5 Department of Pediatrics, University Hospital, Clermont-Ferrand, France; 6 Department of Obstetrics, University Hospital, Clermont-Ferrand, France; Cincinnati Children's Hospital Medical Center, United States of America

## Abstract

Mammalian airways are highly ramified tree-like structures that develop by the repetitive branching of the lung epithelium into the surrounding mesenchyme through reciprocal interactions. Based on a morphometric analysis of the epithelial tree, it has been recently proposed that the complete branching scheme is specified early in each lineage by a programme using elementary patterning routines at specific sites and times in the developing lung. However, the coupled dynamics of both the epithelium and mesenchyme have been overlooked in this process. Using a qualitative and quantitative *in vivo* morphometric analysis of the E11.25 to E13.5 mouse whole right cranial lobe structure, we show that beyond the first generations, the branching stereotypy relaxes and both spatial and temporal variations are common. The branching pattern and branching rate are sensitive to the dynamic changes of the mesoderm shape that is in turn mainly dependent upon the volume and shape of the surrounding intrathoracic organs. Spatial and temporal variations of the tree architecture are related to local and subtle modifications of the mesoderm growth. Remarkably, buds never meet after suffering branching variations and continue to homogenously fill the opening spaces in the mesenchyme. Moreover despite inter-specimen variations, the growth of the epithelial tree and the mesenchyme remains highly correlated over time at the whole lobe level, implying a long-range regulation of the lung lobe morphogenesis. Together, these findings indicate that the lung epithelial tree is likely to adapt in real time to fill the available space in the mesenchyme, rather than being rigidly specified and predefined by a global programme. Our results strongly support the idea that a comprehensive understanding of lung branching mechanisms cannot be inferred from the branching pattern or behavior alone. Rather it needs to be elaborated upon with the reconsideration of mesenchyme-epithelium coupled growth and lung tissues mechanics.

## Introduction

Many conditions lead to ramified shapes in nature. Strikingly, tree-like structures are shared by multi-scale inanimate (snowflakes, bolts of lightning, river basins, viscous fingering etc.) and living systems (trees, vasculatures, mammalian airways, exocrine glands, etc). They are basically encountered when something is growing or flowing in a field from a punctual source to a larger area or volume and vice versa. Although the physical rules involved in these processes are not univocal, the shape of a free-morphing flow is expected to evolve toward a global minimization of flow resistance and/or a maximization of spreading [1]. The tree-like structure of the adult human airways exhibits such striking geometrical properties: the homothety ratios for length and diameters of deep bronchi allow a near-optimal minimization of the airflow power loss [2], [3]. However, the mammalian airway geometry is not shaped in time by airflow to achieve a near-optimal tree. Furthermore, how physical mechanisms contribute to lung branching morphogenesis and how they are connected with lung bud signaling networks remains an open question [4], [5], [6].

During development, airways are generated by the repeated branching of a fluid-filled epithelial tree into the surrounding mesenchymal cell mass. The airways architecture is set up during the pseudoglandular stage (embryonic day (E) 9.5 to 16.5 in mouse). At the beginning of this stage, a primary bud lined with a monolayer of epithelial cells individualizes from the primitive esophagus and extends in the adjacent mesoderm. The first epithelial branching events also split the lung mesoderm establishing a species-specific lobulation pattern. In the mouse this consists of four lobes on the right (cranial, middle, accessory and caudal) and one on the left. Subsequently, the repeated branching of the fluid-filled epithelial tubules occurs into the surrounding mesoderm within each lobe and generates a tree-like structure composed of thousands of branches (for review see [7]).

Recently the complete branching pattern of the mouse airways has been modelized [8]. The bronchial tree is generated by the iterative use of only three elementary modes of branching: domain branching (forming rows of bristles), planar bifurcations and orthogonal bifurcations (so that the four granddaughter branches are arranged in rosette) [8]. Also, it has been proposed that each mode is controlled by a combination of discrete patterning operators (or “subroutines”) that could be genetically tractable: a domain specifier, periodicity generator, bifurcator, rotator and branch generator [8]. The three elementary modes of branching are used repeatedly along the different lineages through only three distinct coupling schemes. Therefore it has been inferred that the complete branching programme of the mouse lung development could rely on the repeated use of “subroutines” that are called at specific times and positions by a “master routine”. However, the genetic basis of this putative branching programme has not been demonstrated to date. Indeed, the main signaling components and cellular behaviors that promote airway architecture are still poorly integrated at the whole organ level [9]. Furthermore, the branching pattern has been described independently of the surrounding tissue geometry, although the mesenchymal-to-epithelial crosstalk is critical in terms of signaling and mechanics in the lung [7], [9] as in other branched organs [10], [11]. The branching architecture per se is thought to determine specific aspects of the lung lobe design [8]. The central scaffold, hence the overall shape of the lobes, are generated by domain branching, while planar bifurcations form the edges and orthogonal bifurcations creates the lobe surfaces and fill the interior [8]. However, several developmental disorders (for example: fetal pleural effusion, isolated congenital diaphragmatic hernia, bell-shaped thorax and rare fetal intrathoracic tumor) suggest that the overall lung design is dramatically sensitive to the pleural fluid pressure/volume, the chest growth and the available space in the thorax [12], [13], [14].

Refining the whole *in vivo* 3-D structure of the mouse right cranial lobe (RCr) through E11.5 to E13.5, we show that the overall lobe design fit the boundary geometry in the chest, while the bud outgrowth locally emboss the lobe surface. The local dynamics of the mesenchyme promotes differential branching rates and induces morphological changes in the tree architecture. Beyond the first generations, the *in vivo* branching pattern is less rigidly stereotyped than previously described. Despite numerous spatial and temporal variations, two morphogenetic features remain highly consistent: the new buds continue to spread homogeneously in the mesenchyme and the growth of the bronchial tree and the mesenchyme cell mass is very strongly correlated at the whole lobe level.

## Results

### The 3D development of the mouse right cranial lobe

**Figure 1:**
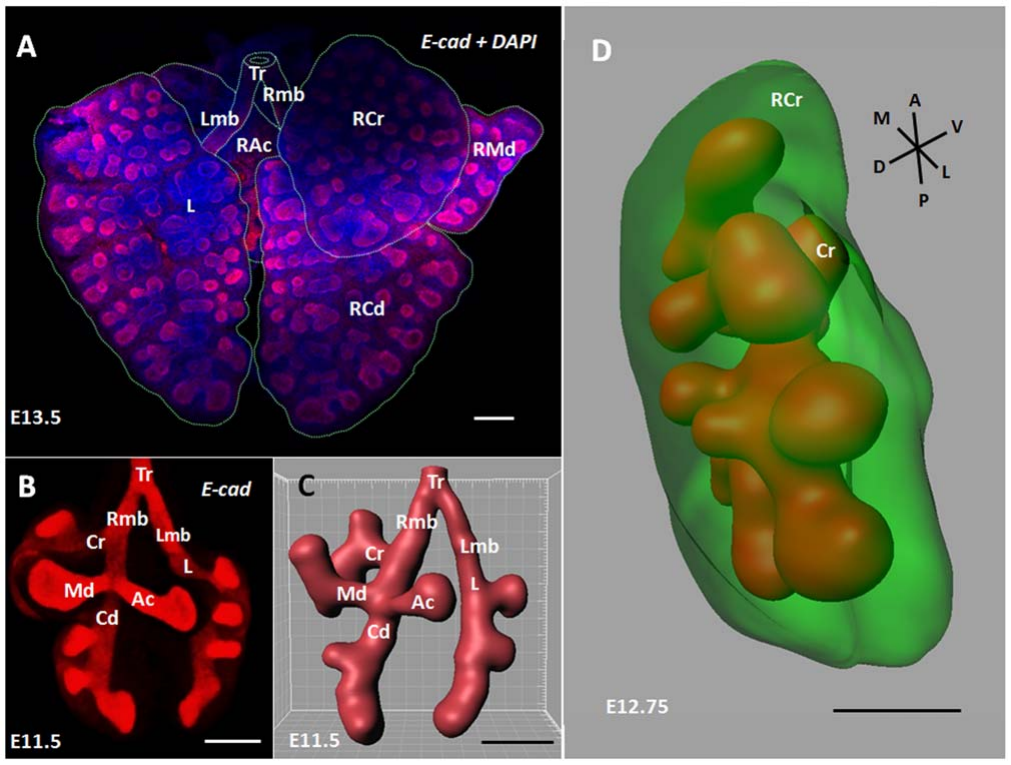
Three-dimensional reconstruction of right cranial lobe full structure. (A) Dorsal view of a whole mount mouse lungs at E13.5 immunostained for E-cadherin (red) and counterstained with DAPI (bleu), showing both the airway epithelium architecture and the shape of the surrounding mesenchyme. Dotted lines show the trachea (Tr), the right (Rmb) and left (Lmb) main bronchi, the right cranial (RCr), right middle (RMd), right accessory (RAc), right caudal (RCd) and left (L) lobes. (B) Ventral view of a whole mount mouse lung immunostained for E-cadherin at E11.5 to show the epithelial tree. Cr, Md, Ac, Cd and L bronchi give rise to the related lobes. The unwanted background around the buds is a limiting factor to perform quantitative analysis. The procedure we developed (see Material and methods) allowed a highly precise 3D visualization of the bronchial tree (C) and the surrounding mesenchymal cell mass (D), with respect to their in vivo relationships. A, anterior; P, posterior; M, medial; L, lateral; D, dorsal; V, ventral; Scale bar: 200 µm.

Because the morphogenesis of the lung mesenchyme and epithelium (Fig. 1A) cannot be monitored in living mouse embryos with the current techniques, we developed an original procedure to reconstruct the full RCr lobe structure with respect to the *in vivo* intrathoracic environment of the lobe (Movie S1). Briefly, the first step of the 3-D reconstruction relied on tomography using histological sections stacks that were performed on the fetuses' thorax (hence the preservation of lung environment). Then stacks alignments were computed and each image was semi-automatically segmented to focus on the lung epithelial tree and mesenchyme (see Material and Methods for further information). We chose to restrict the analysis to one lobe assuming that the branching mechanisms are likely to be similar in each lobe. We did not opt for a classical immunostaining procedure because unwanted background limited the quantitative analysis (Fig. 1B). Using twenty E11.25 to E13.5 wild-type CD1 embryos we generated noise-free 3-D images of the RCr lobe, allowing very precise volumetric visualization of the bronchial tree (Figure 1C) and the lung mesenchyme (Fig. 1D and Movie S2), as well as efficient quantitative analysis of the structure (see below).

### The RCr lobe design is primarily shaped by its dynamic relationships in the thorax

**Figure 2:**
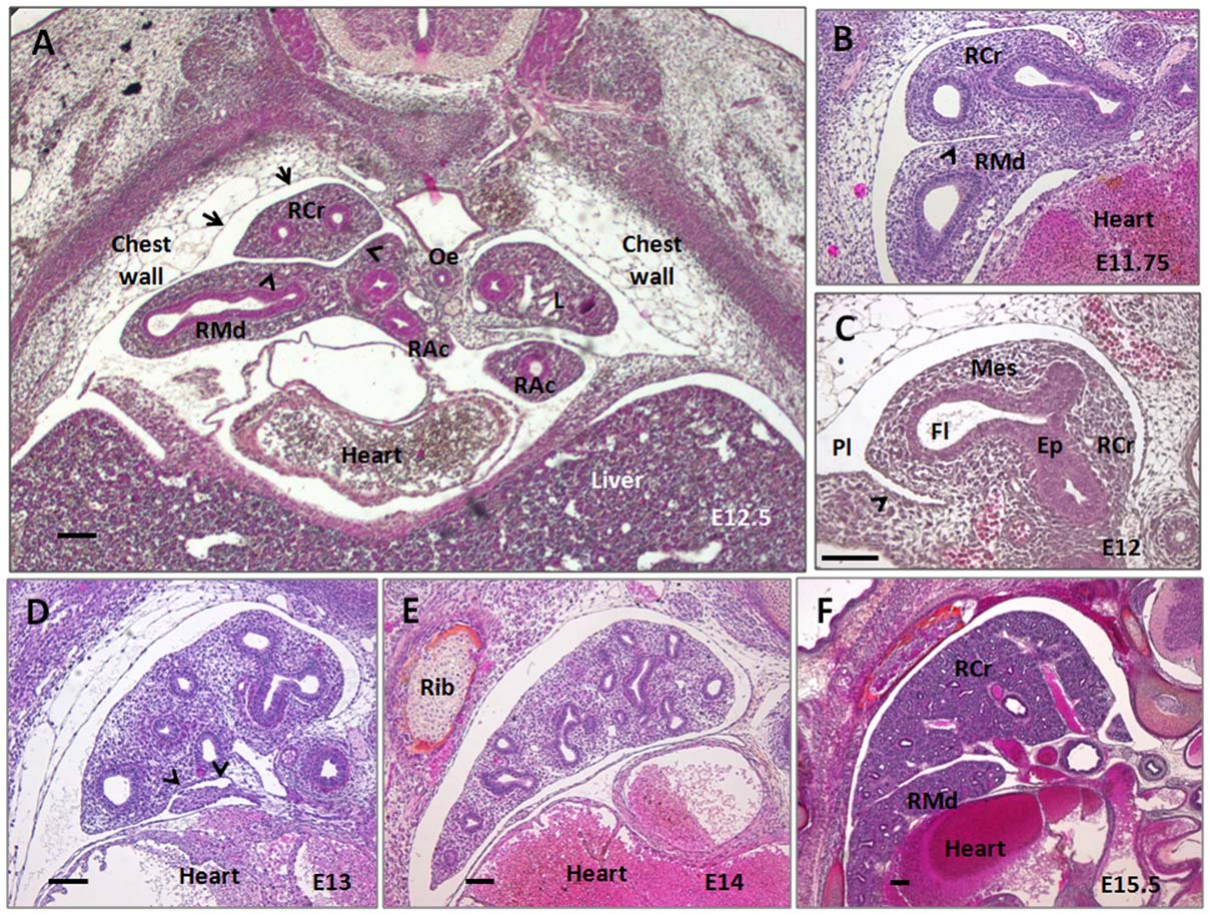
Lung lobe packing in the mouse fetus thorax. Transversal sections of the thorax are performed at the embryonic day indicated and stained with HPS to show the in vivo relationships of the mouse lung. (A) During the pseudoglandular stage, the size and shape of the lung anatomical cavity is mainly constrained by dense tissues (chest wall, liver) or cavities under pressure (heart and larges vessels). RCr develops competing interface with RMd lobe (arrow heads) and slightly imprint the looser tissues of the chest wall (arrows). (B–F) Magnifications show that the RCr lobe surface fits the shape of the surrounding tissues, even if the parietal and visceral mesotheliums are not in strict abutment. The RCr lateral edge is tightly embedded between the chest wall and the heart/RMd. Ep: lung epithelium, Mes: lung mesenchyme, Pl: pleural cavity (outlined by the visceral and parietal mesothelium), Fl: luminal fluid; scale bar: 100 µm.

The current paradigm [8] proposes that each branching mode create specific aspects of the lobe's design. During the pseudoglandular stage, the developing lung was found to be tightly embedded in the surrounding tissues and organs: mainly the chest wall, heart, large vessels and liver (Fig. 2A). Basically, the design of the right cranial lobe (RCr) between E11 and E16 fitted the shape of its anatomical cavity in a dynamic manner (Fig. 2A–F, Movie S1). The relationships could be very narrow, where RCr nearly abutted the facing tissues. The spacing between visceral and parietal mesothelium moderately increased in some other sites (especially lobes edges), but the overall RCr shape remained congruent with the design of the pleural cavity (Fig. 2 A–F). Rcr also developed competing interfaces with the neighboring lobes (right middle and right caudal lobes). Furthermore the lobe slightly imprinted the surrounding soft tissues of the chest wall (Fig. 2A).

**Figure 3:**
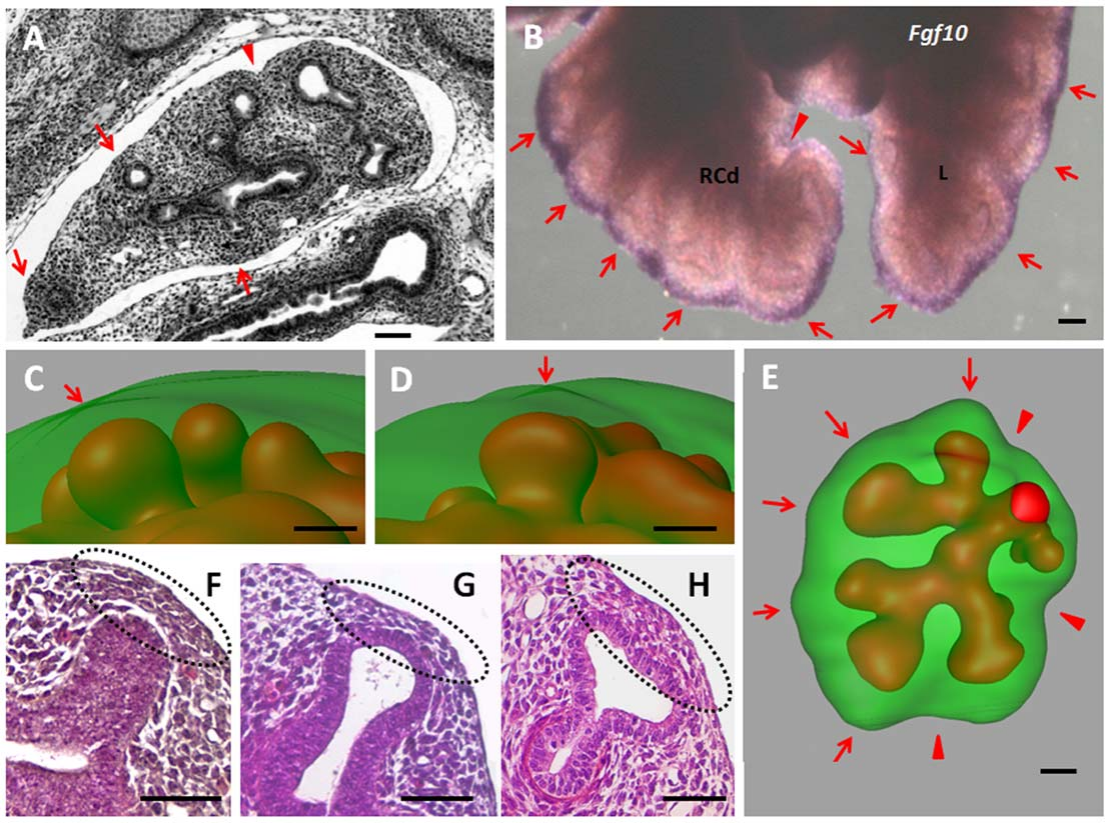
Local bud-induced deformations on the lobe surface. (A) Transverse section of RCr lobe at E13.25 showing bumps (red arrows) and grooves (red arrow head) on the lobe surface, (B) The characteristic Fgf10 expression pattern at E13.25 in the sub-mesothelial mesenchyme outlines regular curves along the lobe edge. (C–E) Three-dimensional reconstructions of RCr lobe at E12.5 to show slight bumps in front of the enlarging buds. (E) Larger and transient grooves appear around group of branches (red arrow heads) whereas small bumps face bud tips or branching sites (red arrows). (F–H) As bud grows toward the lobe surface and reaches the sub-mesothelial area the nucleus density increases specifically in the mesenchyme located between the tip and the mesothelium. In the same time window, the bud tip progressively enlarges and the hump on the lobe surface appears. Scale bar 100 µm.

Conversely, lung bud outgrowth tended to emboss the lobe surface, even if the local deformation was slight and did not change its overall shape (Fig. 3 A–H). The bud-induced deformations were best seen along the lobe edges where the humps formed regular waves (Fig. 3B). As the bud tip reached the submesothelial area, the tip enlarged, the nucleus density of the facing mesenchymal compartment increased and the lobe surface deformed (Fig. 3 F–H). Isolated bud tips or branching buds generated only slight humps on the mesenchyme surface, whereas groups of branches created larger grooves (Fig. 3E). However, all these grooves smoothed in time and never promoted additional interlobular fissures.

Together these data indicate that the shape of the lobe surface result from internal and external constraints. The overall design of the lobe is mainly shaped by steric effects in the developing chest, while the bud outgrowth only creates local and transient deformations. Also, these morphological data highlight the complex mechanics of the lobe morphogenesis and suggest that the mesenchyme growth both result from the allometric growth of the lobe in the pleural cavity and the mechanical strains induced by the bud outgrowth. Because the mesenchyme structure at the tip of the enlarging bud is highly reminiscent of tissue compression, local mechanical strains may also contribute to end-branching events.

### The branching architecture is in turn dependent upon the mesoderm morphing boundaries

**Figure 4:**
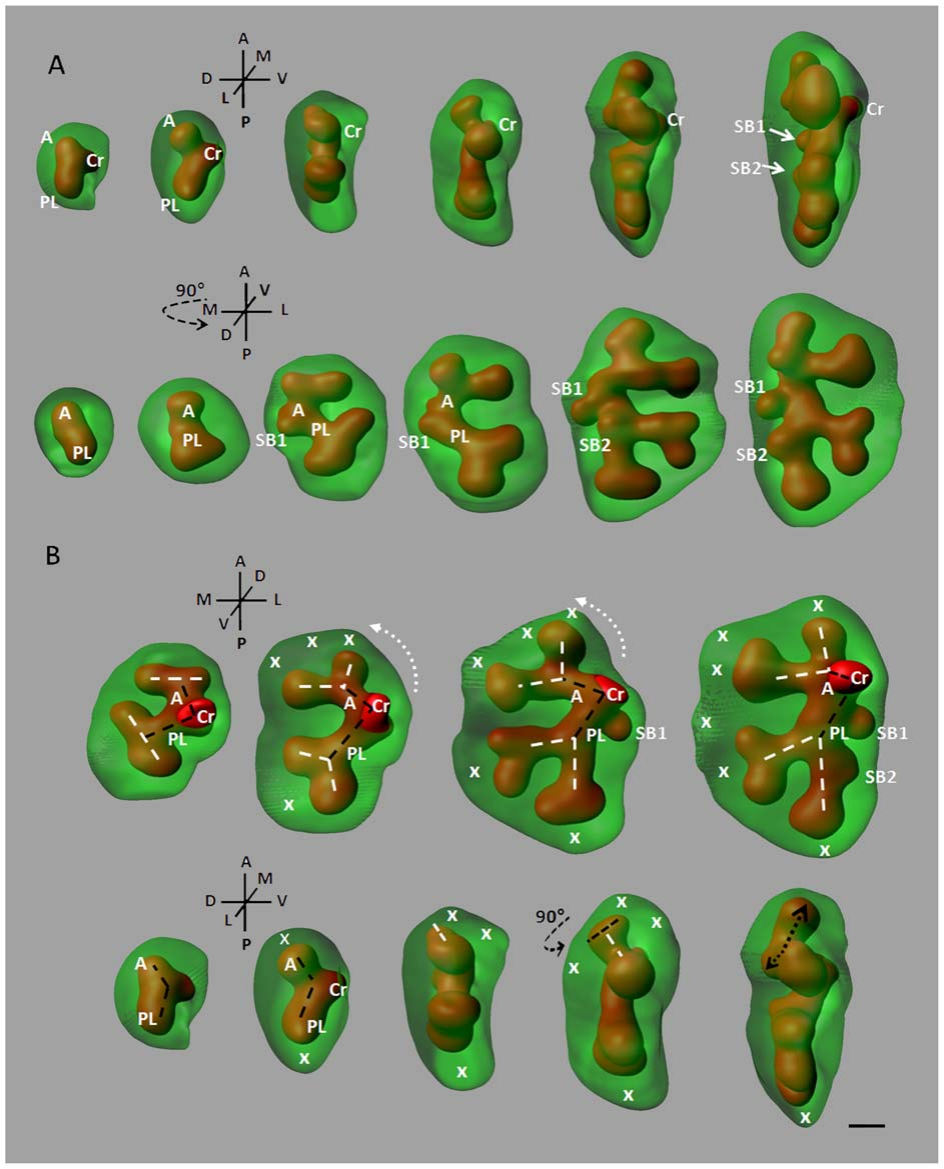
Mesenchyme dynamics effects on the underlying branching architecture. Series of E11.25-E12 RCr 3-D reconstructions, (A) Upper panel: lateral views, lower panel: dorsal views, (B) Upper panel: ventral views, lower panel: lateral views. The RCr lobe quickly elongates along the anterior-posterior axis, first inducing planar bifurcations. The first side-branches (SB1 and SB2) sprout latter, as the mesenchyme thickness increases on the medial and dorsal sides respectively (A). The mesenchyme growth dynamics (white crosses and white dashed arrows) also induces absolute orientation changes of the previously formed branches (black and white dashed lines), rotation at the branching site (rounded black dashed arrow) and differential rate of bud growth (black dashed arrows) (B). Scale bar 100 µm.

Since the overall lobe's design is constrained by the available volume in the chest, either the branching programme has been selected to fit this morphing volume or the branching process adapts to fill the available space in real time. Reconstructing the branching sequence through E11 to E12 (Fig. 4A), we observed that the first bifurcation of the RCr branch (Cr) occurred in a roughly ovoid cell mass of mesenchyme and gave rise to an anterior branch (A) and a posterior-lateral branch (PL). Subsequently the RCr mesenchyme stretched and became ellipsoid (Fig. 4A). Consistent with this, the secondary branches are formed by planar bifurcations following the main growth direction of the mesenchyme. These data demonstrate that domain branching is not used to generate the central scaffold of the RCr lobe and to set its overall shape. Instead, the first side-branch (SB1) sprouted after the secondary planar bifurcation have formed and expanded as the thickness of the lobe increased (Fig. 4A). This later observation suggests that side-branching arise when sufficient space become available around the circumference of a parent branch.

The reconstruction of the early RCr growth showed that its overall shape elongated in a vertical plane and flattened in the horizontal plane. But the local shape of the lobe also underwent differential changes. For example, the initial anterior growth of the lobe was followed by an anterior-lateral expansion of the mesenchyme (Fig. 4B). This growth movement not only promoted planar bifurcations in this direction, but also induced an overall lateral rotation of the underlying branches (up to 90°) (Fig. 4B). This data is consistent with a role for the mesenchyme dynamics in the epithelial tree architecture.

Subsequently, another orientation change occurred at the anterior pole of the lobe where the RCr mesenchyme mostly expanded orthogonal to the preceding main direction (i.e. anterior-lateral versus anterior-ventral). This promoted a rotation of the underlying branching plane: the next generation oriented orthogonal to the preceding bifurcation (Fig. 4B). The new buds grew in opposite directions. The first (OB1) elongated quickly toward the newly expanded area of mesenchyme, whereas the second (OB2) facing the dorsal wall of the lobe expanded slower (Fig. 4B). This result demonstrates that the branching rate is sensitive to the growth rate of the mesenchyme itself and to the morphology of the surrounding mesenchyme.

Together these data indicate the global and local changes of the lobe shape play a role in patterning the branching mode, rate and architecture. Because the lobe design is sculpted by its anatomical relationships in real-time, the branching architecture itself may be structured by these external constraints, rather than being configured early by a branching programme. Also, our results suggest that the mesenchyme growth immediately anticipate the branching events.

### Beyond the first rounds of branching, stereotypy relaxes and variability is the rule

Although the branching pattern looks stereotyped, there are spatial and temporal variations [8]. In the right cranial lobe the architecture of the first three generations is quite stereotyped. But the absolute position, orientation, and sprouting timing of the following branches showed numerous variations.

**Figure 5:**
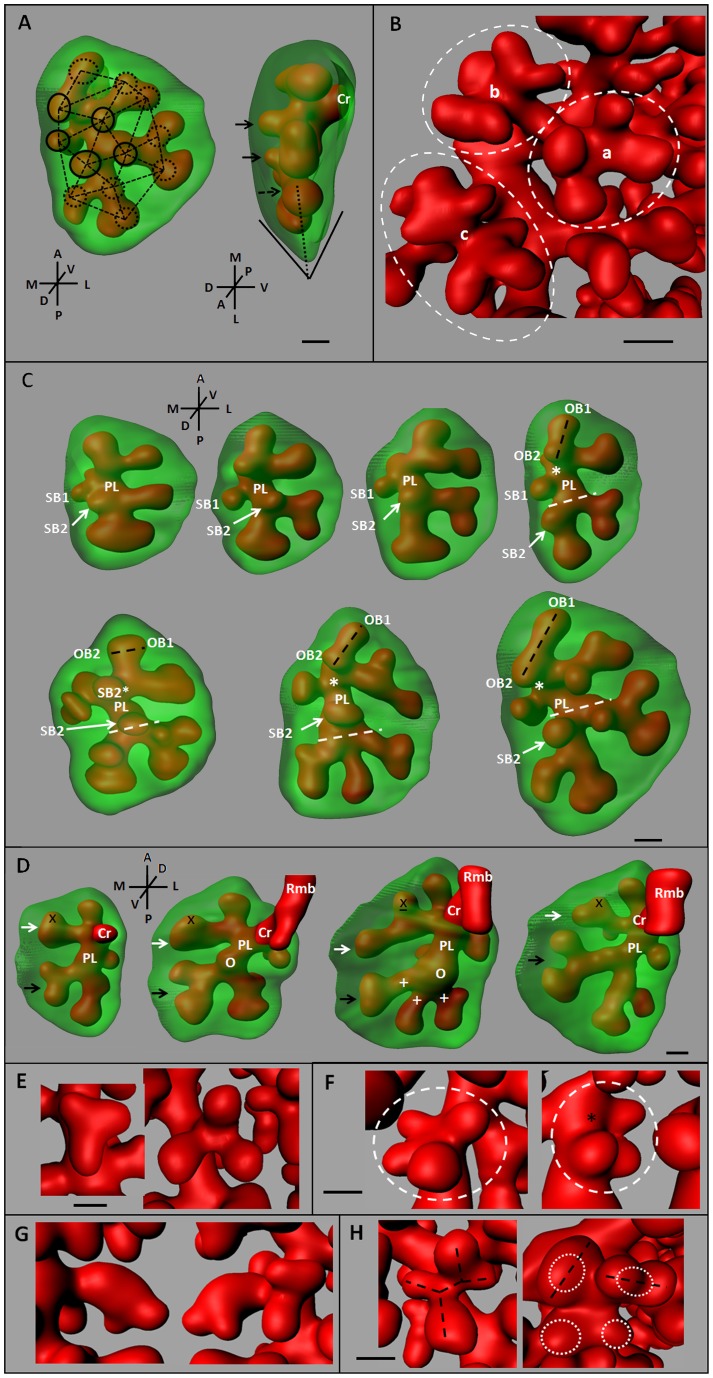
Branching stereotypy and variability. (A) RCr lobe at E12.75. Planar bifurcations fill the angles and occur rigidly in the angle bisector plane (doted line). The side-branches sprout in front of the flat faces (first the medial/dorsal face). They grows in parallel directions and forms rows of bristle (dashed lines), even if they sprout from different parental branches. The side-branches are formed in a proximal-to-distal order (full circles and arrows) from the large perihilar region to the thin edges. Doted circle and arrow depict the next budding sites (see also Figure S1), where mesenchyme is going to enlarge. (B) Branching variations mostly occur in the more open spaces (here the rounded medial face), where a classical rosette (a) co-exist with poorly stereotyped bunches of sprouts (b, c). (C) The sprouting orientation of SB2 (side-branch 2) is highly variable (white arrow). SB2 also originate from variable sites: up, from (dashed white line) or down the PL branching fork. An optional side branch (SB2*) sprouts proximal to SB2 (white star) and modify the bifurcation plane of OB1 and OB2. (D) The PL branch exhibits polymorphic patterns of planar bifurcations or trifurcation (white crosses). The latter originate from large belly also corresponding to an optional-side branch site (white circle). Subtle differences in the mesoderm growth are associated with variable branching rate (white and black arrow). Of interest, the branching pattern also can raise nomenclature confusions: an apparent side-branch (underscored black cross) is indeed generated through end bifurcation (black cross). (E–H) E13.25 RCr lobes showing several morphological branching variants: (E) 3-D trifurcations, (F) rosette directly sprouting from the parental branch, or at the same site, a missing proximal branch (black star) leading to tripod, (G) elbow in the vicinity of another lineage and (H) variable rotation planes twisting the classical rosette (left panel) and the orthogonal bifurcations (right panel). Scale bar 100 µm.

The thin edges of the lobe generated stringent spatial constraints limiting rotation and side-branching. As buds were engaged in the angle, the tip grew toward the vertex until their diameter reached about half of the inter-sides distance, usually not more. As there was no enough space to branch toward the angle sides, all the bifurcations occurred in the bisector plane. Therefore, the planar bifurcation pattern was highly consistent in the edges (Fig. 5A). The first side-branches (SB) developed in medial and dorsal directions after the secondary planar bifurcations have formed and as the lobe thickness increased (Fig. 4A and 5A). Since the lobe thickness gradually decreased from the perihilar region to the edges, the first generations of side-branches sprouted in proximal-to-distal order (Fig. 5A). Remarkably, this order was conserved even if side-branches sprouted from independent parent branches, generating radial rows of unrelated buds (Fig. 5A). Moreover, although the side-branches sprouting sites showed variations (see below), buds were always oriented parallel (Fig. 1D and 5A). Together, arrays of planar bifurcations in the thin edges and “virtual domains” of proximal-distal side-branches indicate that stringent spatial constraints promote the emergence of stereotyped features in the branching pattern. Also, because buds tend to distribute regularly in the opening spaces, they may be sensitive both to the mesenchyme geometry and the neighboring buds position.

Spatial variations mainly occurred where the spatial constraints were looser – namely under the wide-opened borders, flat faces and in the interior of the lobe (Fig. 5B). In these regions, irregular bunches of sprouts were observed (Fig 5B). The earlier variations involved the primary generation of side-branches. The first side-branch (SB1) originated variably from Cr, its daughter PL or from the Cr branching fork (Fig. 5C). The second side-branch (SB2) also sprouted from variable sites: PL or its posterior daughter or the PL fork (Fig. 5C). The absolute anatomical orientation of the budding site was also variable: medial to dorsal. The budding points did not shift along the parental branch over time, as later lungs showed the same range of spatial variability. An optional side-branch (SB2*) was found to sprout prior to SB2 from the Cr-A junction or the A. branch in _∼_40% of cases (Fig. 5C). In this case, OB2 did not dive towards this newly occupied space (i.e. in posterior-dorsal direction) and reoriented medially, demonstrating that a variant lineage can modify the branching orientations of a neighboring lineage (see also Figure S1). The SB2*-associated rotation shift (a neighboring orthogonal bifurcation turn into planar bifurcation) not only point out the tight positional interplay between buds. Because orthogonal bifurcation occurred immediately at the next generation and filled the anterior-lateral expanded mesenchyme (Figure S1), the results also suggest a local-regional coupling between the epithelial buds and the mesenchyme growth.

Temporal variations occurred as early as the second generation after the Cr formed and were very frequent thereafter. They did not involve specific lineages or positions. The bud outgrowth and the branching rates in a lineage were both influenced by those of neighboring lineages and the growth of the surrounding mesenchyme (Fig. 5D). On one hand, subtle growth differences in the same mesenchyme area lead some lineages to get ahead of, or fall behind, a neighboring lineage. On the other hand, once a lineage filled a mesoderm area, the neighboring lineages could not access to this area any longer. This behavior suggests that lineages compete to fill the opening spaces in the mesenchyme and that buds may exert a screening effect on their neighbors.

Only three modes of branching have been shown to account for the entire branching events [8]. However, the RCr lobe branching pattern exhibited several morphological branching variants. The PL branch usually bifurcated into 2 daughters. But in the same littermates, we observed a planar trifurcation originating from a large belly instead of a regular bifurcation fork (Fig. 5D). The location of this swelling epithelium also corresponded to the origin of an inconstant side-branch of PL (_∼_50%) (Fig. 5D). Indeed, the branching pattern was polymorphic at this site, and all of these morphological variants suited distinct mesenchyme designs (Fig. 5D). We not only observed planar trifurcations, but also 3-D trifurcations (10–20% of cases), mostly facing the flat faces of the lobe (Fig. 5E). Some side-branches sprouted from the parental branch in a restricted area forming a rosette-like structure or alternatively a tripod (Fig 5F). In addition, we identified an elbow on a medial side-branch (Fig. 5G). This indicated that branches not only undergo orientation changes after they have formed, but also while growing and branching. Finally, the bifurcation plane was not always orthogonal to the preceding bifurcation to form regular series of orthogonal bifurcation. Various orientations were observed in the interior of the lobe and underside of the flat faces, even more so for the first branching generations undergoing rotations. These variations disrupted the classical rosette structures and led to poorly systematized patterns of variable rotations (Fig. 5B, 5G).

Several of these variations did not occur at specific times and positions, demonstrating that lung epithelium dynamics is more complex than previously thought. Also, because epithelial buds never meet after lineage variations, the results imply the branching mechanisms are locally regulative, so that bud adapt in real-time to the spatial configuration of their microenvironment.

### Despite relaxation in branching stereotypy and local variations in mesoderm design, the growth of the epithelial tree and the mesenchymal compartment is tightly coupled at the whole lobe level

**Figure 6:**
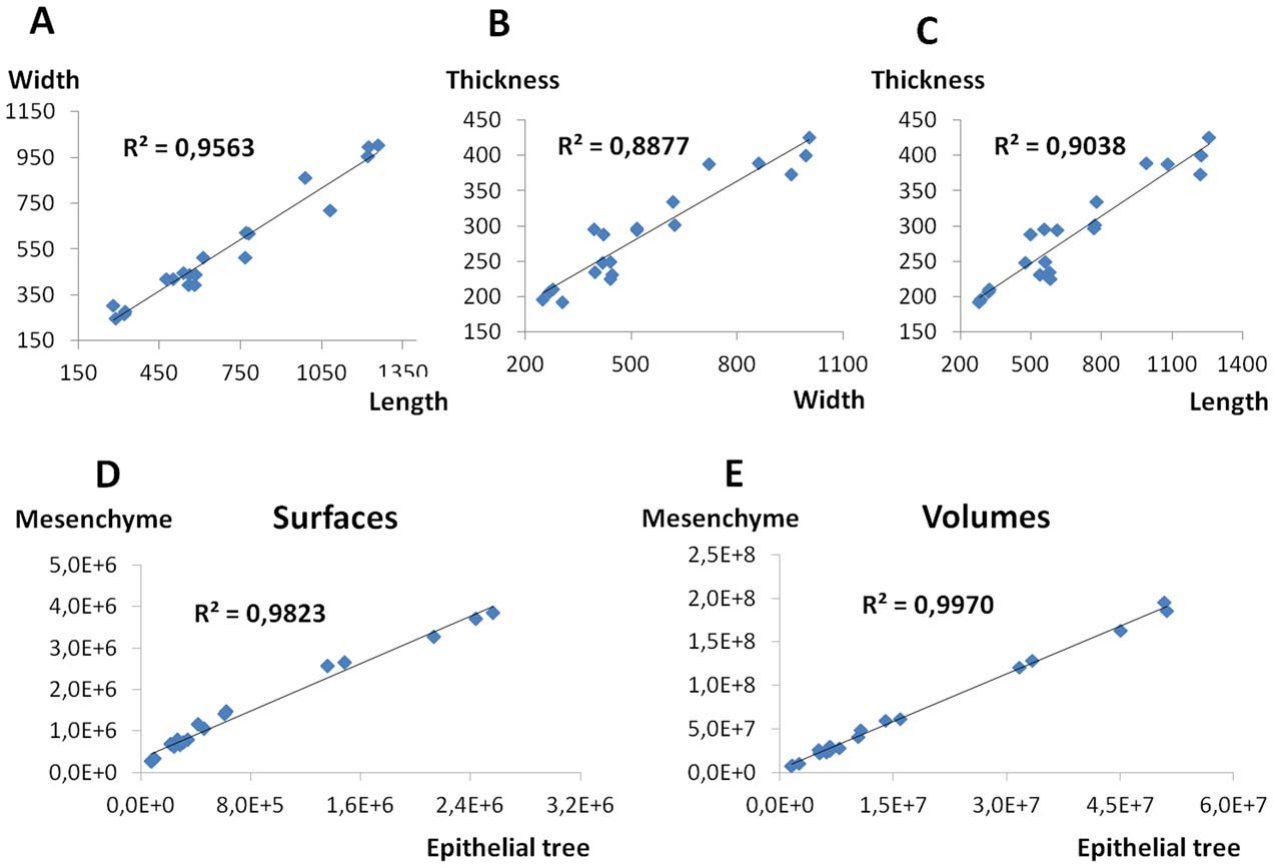
Overall growth coupling of the bronchial tree and the mesenchyme. Graphs plotting the length against the width (A), the width against the thickness (B) and the length against the thickness (C) of a series of E11.25-E13.5 RCr lobes. Using the same specimens, the surface and the volume of the bronchial tree were plotted against the respective surface and volume of the mesenchyme cell mass (E and D). The main dimensions are strongly correlated showing that slight inter-specimen differences occur while the overall shape of the RCr lobe is conserved. In comparison, the overall growth of the epithelial tree and the mesenchyme compartment are very strongly correlated at the lobe level. Number of analyzed specimens: 20. Distances are denominated in µm, surfaces in µm^2^ and volumes in µm^3^.

The overall shape of the RCr lobe is conserved between specimens; however, it is not invariant locally. Within the same littermates, the lobe shape showed focal variations (see Fig. 5D). To quantify the magnitude of these inter-individual variations over time, we plotted the length against the width (Fig. 6A), the width against the thickness (Fig. 6B) and the length again the thickness (Fig. 6C) of each specimen through E11.25 to E13.5. Length, width and thickness represent the largest dimension of the RCr lobe along the anterior-posterior, medial-lateral and dorsal-ventral axes, respectively. The graphs confirmed that length grew fastest followed by width and then thickness. Slight Inter-individual variations were also confirmed: for instance lobes with similar length showed different thickness. Despite variations, the correlation between the specimens' dimensions remained strong (0, 88<R^2^<0,95), demonstrating that the mesenchyme grows focally at somewhat different rates between specimens, with respect to the overall shape of the lobe.

However, the latest measure only took into account the largest dimensions of the lobe following the three anatomical main planes. To refine the global growth dynamics of the epithelium and the mesenchyme at the lobe level, we measured the surface and the volume of the epithelial tree and the mesenchyme for each specimen. Plotting the epithelial surfaces against the lobe surfaces (Fig. 6D) or the bronchial tree volume against the mesenchyme cell mass volume (Fig. 6E), we observed that both where very strongly correlated through E11.25 to E13.5 (r^2^ = 0.98 and 0.99, respectively). Moreover, we found that lobes having similar surface or volume ratio (bronchial tree to mesenchyme) could also have slight differences in branching architecture and mesoderm morphology. Thus, although the RCr lobe shows spatial and temporal variations in the branching pattern and mesenchyme dynamics, and although epithelium and mesenchyme have different shapes, their growths are highly coupled in real-time at the whole lobe level.

### The developing bronchial tree fills homogeneously the available space in the growing mesenchyme

**Figure 7:**
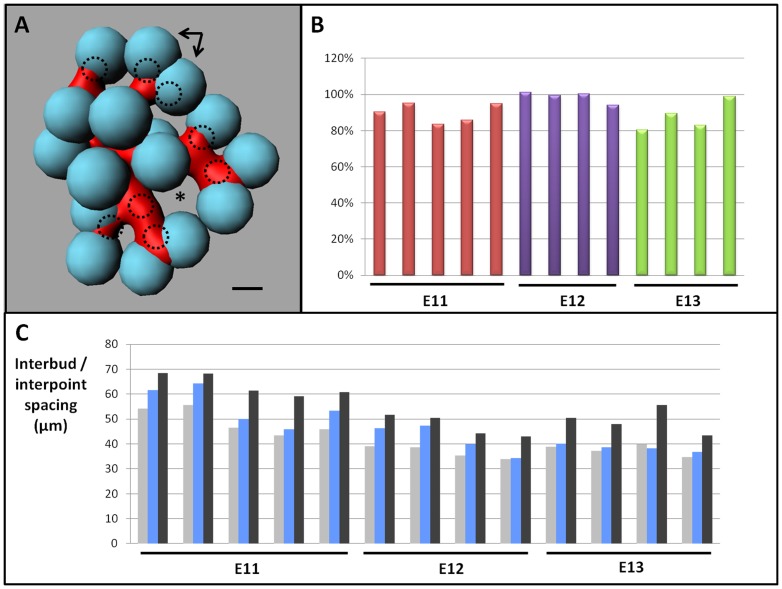
Space filling properties of the developing bronchial tree. (A) Spheres (blue) are allowed to grow at the bud tips until they reach one another and/or the lobe surface. Given these conditions, spheres show only slight differences of diameters. The minimal sphere radius within a lobe is used as in vivo space filling criterion (Kiv). Arrows indicate intricate spheres at site undergoing bifurcation. The gaps between the spheres are predictive of the next branching sites: dorsal side-branches (doted circles) and posterior side-branch (star). (B) Kiv is given as percentage of the optimal value approximated by the best random value BRV (see Material and Methods). Although the best random spacing is geometrically overrated (see Material and Methods), all the Kiv values are higher than 80% of BRV at E11 (red), E12 (purple) and E13 (green). (C) From E11 to E13, Kiv (blue bars) are comprised between the 99th BRV percentile (light grey bars) and the BRV value (dark grey bars), except for one E13 lobe, demonstrating that new bud tips are not distributed at random and tend to fill homogeneously the available space in the mesenchyme. Scale bar 100 µm.

A striking qualitative feature of the bronchial tree was that the branching process continued normally, while undergoing numerous local variations in timing, branching configurations or morphologies in CD1 wild type background. Buds never conflicted with one another. Rather they were arranged in regular arrays through the lobe's volume (Fig. 5A). To determine the homogeneity of the new buds distribution, we reasoned we could compute spheres centered at bud tips and allow them to grow until their surface reaches another one or the mesenchyme boundary (Fig. 7A). We defined a space filling criterion (K) as the radius of the smallest sphere in a lobe (See Materials and Methods). Then, we compared the criterion measured in each lobe (Kiv) to the “best random value” (BRV) we obtained after 100.000 configurations at random in the matched geometrical conditions (Movie S3). Although the design of the random distribution led to slightly overrate the optimal spacing in the lobe volume (See Materials and Methods), Kiv fell in the range from 80% to 105% of BRV through E11 to E13 (Fig. 7B). Furthermore, Kiv were higher than the 99^th^ percentile of BRV – except for one E13 RCr (Fig. 7C), demonstrating that bud tips are not distributed at random (p = 0.01). Together, these results imply that steric effects play a role in lung bud distribution and that new bud tips tend to spread homogeneously in the mesenchyme volume. Also, because the gaps between the spheres are predictive of subsequent budding sites (Fig. 5A, 7A and Figure S1) and matched with the new available spaces in the mesenchyme, the results indicate that the mesenchyme growth slightly anticipate bud branching. Together these data suggest that news buds may adapt in real time to the surrounding spatial configuration, with only a slight time-lag on the mesenchyme dynamics.

## Discussion

### Reconsidering the branching pattern in light of the lung epithelial tree environment

The branching programme of the mouse lung development has been elaborated using a morphometric analysis of the 3-D structure of the epithelial tree alone in a series of fixed lungs [8]. Performing a 3-D reconstruction of both the lung epithelium and mesoderm *in vivo* architecture, we identified that both the bud outgrowth and the branching rate are sensitive to the way the surrounding mesenchyme is morphing. Yet the lobe boundaries are not free to morph in the developing chest. Basically, the lung is packed between the chest wall and the other intrathoracic organs. Strikingly, the shape of the lobe is always congruent with the shape of the surrounding tissues and organs, whether they are in abutment or just close. Because of the difficulty of accurately measuring pleural liquid and pleural surface pressure during the early lung development, very little is known about the role of fetal pleural space in morphogenesis. In the third trimester, the transpulmonary pressure is about 2 to 2.5 mmHg outside of fetal breathing movement periods in the fetal sheep [15]. This is due to a positive intratracheal pressure of _∼_2.0 mmHg and a negative pleural pressure of _∼_0.5 mmHg [15]. Thus, if similar during the pseudoglandular stage, the boundary conditions are likely to generate a pressure gradient in the pleural space [16] and to promote the growth of the lobe surface toward the chest wall. It is also clear from the lung organotypic culture experiments that lung tissues (both the epithelium and the mesenchyme) have intrinsic growth potential (see [17] for example). Together the growth and shape of the mesenchyme are likely to result both from internal driving forces (such as the bud outgrowth and the mesenchymal cell proliferation mediated by the epithelial-mesenchymal signaling crosstalk) and external driving forces (the available space in the chest, the competing boundaries with the surrounding tissues and organs and the allometric growth of the pleural cavity). But based on our results, we propose that the allometric growth and the shape of the pleural cavity act as main drivers on the lung lobe design. As previously suggested [18], we also anticipate that the moving boundaries effects of the thoracic cavity on the lung development are mediated by the transpleural pressure.

### A plausible role for mechanical strains in tip splitting

The bud outgrowth progressively deforms the mesenchyme and enlarges the bud tip. The closer to the mesothelium the outgrowing bud, the larger the bud tip and the larger the mesenchyme bump on the lobe surface. In this case, the mesenchyme structure at the bud tip is reminiscent of tissue compression, suggesting that buds gradually push and compress the surrounding mesoderm. Two histologically distinct regions have already been reported in the mesenchyme: the sub-epithelial mesenchyme (SEM) and the sub-mesothelial mesenchyme (SMM) [19]. The former is a dense layer of flattened/stretched cells wrapped around the epithelial tubes. The latter is a looser network of non-oriented cells filling the space between the SEM and the mesothelium. SEM results from Fgf9-induced mesenchymal cell proliferation [19], [20] and Fgf10-positive cell migration around the tubes [21]. SMM proliferation is also stimulated by FGF9 [19]. Our results show that the nuclear density is not homogeneous in the sub-mesothelial compartment and increases specifically between the sub-mesothelial bud tips and the bumps on the lobe surface. Thus, we hypothesize that the sub-mesothelial mesenchyme is compressed by the growing bud, and in turn opposes increasing passive and/or active mechanical resistance to the bud tip. Supporting this hypothesis, the kinetics of bud outgrowth in cultured lungs has shown that buds stop once reaching the sub-mesothelial area, while enlarging just before branching [17].

### Order and variations

As early as 1962, Weibel and Gomez noticed that the human lung architecture exhibits both an irregular pattern of dichotomous branching and harmonic features [2]. Of interest, they proposed to neglect irregularities at first, assuming that the reduction of the true airways to a system of regular branching will prove useful for some overall aerodynamical consideration if appropriate precautions were taken [2]. Further physiological studies confirmed the relevance of this anticipation [3], [22], [23], and branching irregularities have only recently been taken into account in functional modeling [24]. In the same way, lung branching has been thought to be stereotyped for a long time [25], [26], [27]. This led to elaborate on a very concise paradigm for the complete branching process [8]. Remarkably, deviations from stereotypy have also been reported in the same study. Therefore, it has been proposed that variations and errors – some of them occurring up to the frequency of the normal event – could identify imprecise steps in the global branching programme [8]. But one can ask if a branching error approaching the frequency of the normal event still should be called “error.” The branching process shows very low variability during the two or three rounds of branching after the cranial branch has formed. Thus, the early branching process looks quite stereotyped. Also, the first branches are generated through end-bifurcations, demonstrating that the basic scaffold of the RCr lobe is not set by domain branching. Indeed, the branching architecture analysis is highly sensitive on the screen capture's timing of the branching events. After E12-E12.5, both temporal and spatial variations are frequent. The first dorsal side-branches sprout from variable sites and even branches. The side-branch daughters usually bifurcate but also trifurcate (_∼_10–20%). Of interest, early trifurcations occur up to 20% of cases in the human left lung [28]. In addition, the side-branch daughters show variable rotation planes. However, because buds tend to homogeneously fill the available space in the mesenchyme, higher order arrays emerge and the branching pattern still can look like an ordered mess. Our results demonstrate that the branching pattern is highly sensitive to steric effects. On one hand, the mesenchyme geometry is likely to account for the stereotypy of the early planar bifurcations (mesenchyme edges are filled by planar bifurcation). On the other hand, lineages exert reciprocal spatial interactions: the rotation planes of the bifurcations adapt according to the configuration of the neighboring lineages, and the new buds compete to fill the opening spaces in the mesenchyme. Only a few degrees of freedom in the mesenchyme geometry or in buds sprouting are sufficient to promote spatial or temporal variability. An adverse effect of this variability in a wild-type background could be to complicate further phenotypic analysis of mutants. It is now clear that stringent precautions should be taken to avoid confusing conclusions. For example, null mutations in sprouty 2 (*Spry2*) have been suspected to have local and subtle effects on some side-branches by shifting their sprouting site (a phenotype that was named “shifty”) [8]. Our data show that such shifting events also occur in CD1 wild-type background: the sprouting site of much of the early side-branches can vary along a parental branch and can also shift to the daughter. Moreover, optional lineage in wild-type background may also confuse the concept of additional lineages in mutants.

### The lung branching programme

The current paradigm is based on branching stereotypy and canonical branching rules. A master programme is proposed to set a coupling scheme for each lineage early, and then allows each lineage to proceed through its sequence independently [8]. This raised interesting speculations on the amount of patterning information which could be required to encode the programme. The iterative use of elementary, evolutionary conserved [29] branching modules could alleviate the genetic coding of the programme [8]. However, calling slave routines at specific times and positions throughout the lung represent a complex input of local patterning information. Moreover, the numerous local variations make the programme very intensive to encode, probably more than genetically plausible. Indeed, the variability of the branching process challenges the idea of a predefined programme and supports a conceptual shift toward a local, real time regulation to adapt continuously to the available space.

### Beyond master and slave programme

Metzger and collaborators proposed two working hypotheses: 1) the branching process occurs randomly to fill the available space or 2) the branching process is simplified by the repeated use of elementary branching mechanisms. Our data strongly support the idea of a real time space filling process. Alternatively, we propose that the process is not “random”. Rather, the mesenchyme geometry and the steric effects of the bud seem to exert drastic constraints on branching morphology and timing (buds adapt and compete to fill the gaps). Tissue geometry has already been shown to determine the branching pattern in organotypic cultures of mammary epithelium [30] and is thought to support long range signals by sculpting gradients of morphogens and mechanical strains [31]. This framework is very attractive because it could explain both why the growth's interplay is regulated at the whole lobe level and how each bud “feels” individually the available space. To date, several branching models have been based on key morphogen diffusion [32], [33], [34], [35], [36], [37]. But boundary conditions are usually simplified for convenience, and diffusion coefficients fail to accurately simulate the anisotropy of the *in vivo* conditions. For example, it has been suggested that mechanical loading modulates morphogen distribution [38]. Although the importance of mechanical forces in lung morphogenesis has been known for a long time [39], [40], the way they contribute to the lung branching mechanisms is still poorly understood [4], [7]. Recently, endogenous patterns of mechanical strains have been shown to play a role in the epithelial branching process [41]. Also, the mechanical role and behavior of the epithelium [42] and the mesenchyme [43], [44] are raising increasing interest. Again, it is clear that the mesenchyme is a histologically and mechanically heterogeneous compartment. For instance, it contains migrating and differentiating cells, such as parabronchial smooth muscle cells [18] that can quickly modulate the strain map. Moreover, the vasculature architecture has been shown to influence the branching pattern [45]. According to this, we also found that branching was delayed in the vicinity of the perihilar region, where the main vessels get into the lobe (data not shown).

Physics and biology promise to converge rapidly in the field of lung development, as illustrated by a recent study [46] demonstrating that the shape of the buds is correlated with mitotic spindle orientation control through the differential activation of the Ras-Erk pathway along the tubes.

### Conclusion

The 3-D reconstruction of the *in vivo* E11.25–13.5 whole RCr lobe architecture highlights the tight coupling of the epithelium and mesenchyme growth, both at the bud scale and the whole lobe level. The branching process is constrained by the boundary geometry of the mesenchyme that fits itself within the shape of the lung anatomical cavity. We show that the lung branching pattern cannot be parsed independently of the mesoderm dynamics and vice versa. Because steric effects appear to play a critical role in space filling, the stereotypy of the branching pattern is associated with tight spatial constraints, as during the first rounds of branching. Subsequently, although stereotypy relaxes, buds continue to homogeneously fill the available space in the mesenchyme. An important challenge is now to determine how buds feel the available space in real-time, and how the global dynamics of the epithelium and mesenchyme are regulated. Based on our data, we propose that both the morphogens diffusion map (R. Clement, P. Blanc et al., accepted) and the mechanical strains map (P. Blanc et al., in preparation) need to be integrated in the lung morphing geometry to achieve a comprehensive model of the branching process. Whatever the lung branching paradigm will be based on, it will have to deal with both stereotypy and variations.

## Materials and Methods

### Mouse studies

All mice were maintained in plastic cages with *ad libitum* access to food and water. The animal procedure followed the French and EU guidelines with the approval of the local ethics committee of animal care and use (Animal Experimentation Ethics Comity of Auvergne -CEMEAA- # B63–175).

### Whole mount antibody staining with anti-E-Cadherin

Lungs were stained as previously described (for full procedure see [8]) with an additional step of DAPI counterstaining of one hour at room temperature. Lungs were then imaged on LSI Leica®.

### Whole mount in situ hybridization

Mouse embryos (CD1) were dissected in cold (4°C) PBS and lungs were immediately fixed in 4% paraformaldehyde/PBS (wt/vol) at 4°C, with gentle rocking for 1 hour. Fixed lungs were washed in PBS for 5 min at room temperature, with gentle rocking. Lungs were then dehydrated by washing once in 25% methanol/PBT (PBS with 0.1% (vol/vol) Tween-20), once in 50% methanol/PBT, once in 75% methanol/PBT, and twice in 100% methanol. Dehydrated specimens were stored at −20°C in 100% methanol (some of them for 10 months before use without obvious deterioration of staining results). Following steps were carried out into 2 ml RNAse Free tubes and in 1 ml of reagent, at room temperature with gentle rocking, unless otherwise stated. On day 1, dehydrated lungs where rehydrated through an inverted methanol/PBT series (5 min washes in each of 75%, 50% and 25% methanol/PBT, followed by 2×5 min washes in PBT). Lungs were permeabilized 5–6 min (depending on the size of the lungs) with 10 µg/ml proteinase k/PBT and digestion was stopped by washing 5 min with 2 mg/ml glycine/PBT. Specimens were washed 2×5 min in PBT, refixed for 20 min in 0.2% glutaraldéhyde/4% paraformaldehyde, and washed again 2×5 min in PBT. Lungs were then incubated in hybridization solution (50% formamide (vol/vol), 5× SSC, 1% SDS, 0.1 mg/ml of yeast RNA, 0.05 mg/ml of heparine) for 1 h at 65°C, and then they were incubated overnight at 65°C in a 1 µg UTP-DIG labeled probe/300 µl hybridization solution. The cDNA used as template for the Fgf10 riboprobes (pKS-mFgf10) was kindly provided by Dr. S. Bellusci. On day 2, lungs were rinsed twice (10 min and 30 min) at 65°C with washing solution n°1 (50% formamide (vol/vol), 5× SCC, 1% SDS), 30 min at 65°C with washing solution n°2 (50% formamide (vol/vol), 2× SCC) and 2×5 min with TNT (100 mM Tris pH 7.5/150 mM NaCl/0.1% Tween-20). Blocking was performed through 1 hour wash in TNB (0.5% (wt/vol) Blocking Reagent (Perkin Elmer)/TNT). Lungs were incubated 2 hours with the Antidigoxigenin-AP Fab fragments (Roche) diluted 1∶1000 in TNB. A series of washes with TNT was carried out (5 min –10 min –15 min and 3×20 min). Lungs were then washed 3×5 min in NTMT (100 mM Tris pH 9.5/100 mM NaCl/50 mM MgCl2/0.1% Tween-20). Specimens were incubated overnight with a 20 µl NBT-BCIP (Roche)/1 ml NTMT solution. Staining reaction was stopped by removing the mix above and adding 1 ml of cold (4°) PBS. Finally lungs were transferred in 4-well plates (Nucleon Surface NUNC) and imaged using SZX12 Olympus.

### 3D reconstructions of mouse fetal lungs

Mouse Embryos were collected between embryonic day (E)11.25 and E13.5 and were dissected in cold (4°) PBS (head and caudal end below the liver were removed). For histological analysis, whole thorax were fixed in AFA (alcohol + formalin + acetic acid) for 24 to 48 hours at room temperature, embedded in paraffin, cut at 5 µm and stained with HPS (hematoxylin-phloxine-saffron). The HPS stained section series were arrayed on microscope slides, snapshots were taken at low magnification (x4) and images were stacked using MetaMorph software. Image stacks were aligned using FIJI software. All the relevant structures (epithelium tubules and mesenchyme cell mass) were segmented semi-automatically to ensure a complete thresholding of the noise (FIJI software). 3D reconstructions of the full cranial lobes were performed on Bitplane Imaris 7.3.1.

### Space filling criterion calculation

Each RCr lobe is characterized by a number of bud tips (Nx) in a volume (Vx). The tip-to-edge and intertip lengths were examined in each specimen to assess the bud tip distribution in the RCr lobe volume. As the bud-tip is roughly hemispherical, we defined the center of the related sphere as the barycentre (Bi) of the tip. The minimal tip-to-edge (Ei) and the intertip (Ti) lengths were measured for each Bi in a lobe. Then Di = min (Ti/2, Ei) were calculated in the lobe. This distance gave an *in vivo* space filling criterion (K_iv_ = min Di) which was calculated in each lobe. Then we computed 100.000 distributions at random for all (Nx,Vx), using Matlab® software (see Figure S2). For each distribution n we calculated K_n_ and the “best random value” (BRV  =  max K_n_). Thus, BRV was an indicator of the maximal spacing of Nx points in the volume Vx, hence of the homogeneity of the points distribution. Following K definition, BRV converged toward a theoretical optimal distance (i.e. the best spacing). However, BRV was expected to slightly overestimate this optimal value, because the Nx points were allowed to distribute at random everywhere in Vx, while the epithelial tree generated a dead space *in vivo* (i.e no bud tip located inside the tree). Finally, Kiv was compared with BRV for each specimen.

## Supporting Information

Figure S1 **Branching sequence of the early dorsal side-branches.** Panel A and B reproduce the E12.75 RCr lobe of figures 5A and 7A respectively. Panel C show a E13.25 RCr lobe. The full white circles depict branches that have already formed at E12.75 and dashed white circles show that the topography of the news buds is consistent with the E12.75 putative budding sites (dashed black circles, panel A). The gap in the meshwork (black star, panel A and B) is filled subsequently by an additional posterior lineage that gives rise quickly to a dorsal branch (white star). Panel C also show the outcome of the SB2* optional lineage. Of note, the orthogonal bifurcation forming “OB1” and “OB2” turn into planar bifurcation and give rise subsequently to ventral and dorsal branches.
(TIF)

Figure S2 **Random series thresholding.** Given N points (the bud tip number) in a RCr volume V, there is a infinite number of configurations of the N positions in V. We computed X configurations at random of N points in V (here a E12.25 RCr lobe), where X was comprised between 1 and 200000. For each configuration, we calculated the space filling criterion K and then extracted the best random value (BRV) of the series. For each X value we repeated this procedure 100 times. X-axis represent the X values and Y-axis the mean BRV. Standard deviation is reported for each BRV mean. Between X = 100.000 and X = 200.000, the BRV value only increases by 1 µm, indicating that the precision gain is about 2% while the number of simulation doubles. This strongly suggests a convergence of BRV to theoretical optimal value.
(TIF)

Movie S1 **Three-dimensional segmentation of the RCr lobe** (**E11.75**)**.** Using FIJI Software, the bronchial tree (red) and the mesenchymal compartment (green) of the RCr lobe are segmented semi-automatically on each slide, allowing subsequent a 3-D reconstruction of the whole lobe structure, with respect to its in vivo shape and relationships.
(WMV)

Movie S2 **Three-dimensional reconstruction of the RCr lobe** (**E13.25**)**.**
(AVI)

Movie S3 **Random distribution test in a E12.75 RCr lobe.** The geometric features of the lobe (the number N of bud tip and the mesenchyme volume V) are used to compute 100.000 distributions at random of N points in V and to calculate the best random value BRV (i.e. the maximal-minimal interpoint distance of the series).
(WMV)
