# Supplementary figures and images for: A Role for Mesenchyme Dynamics in Mouse Lung Branching Morphogenesis
Source: PLoS One. 2012 Jul 23;7(7):e41643. doi: 10.1371/journal.pone.0041643 (PMC3402475; doi:10.1371/journal.pone.0041643)

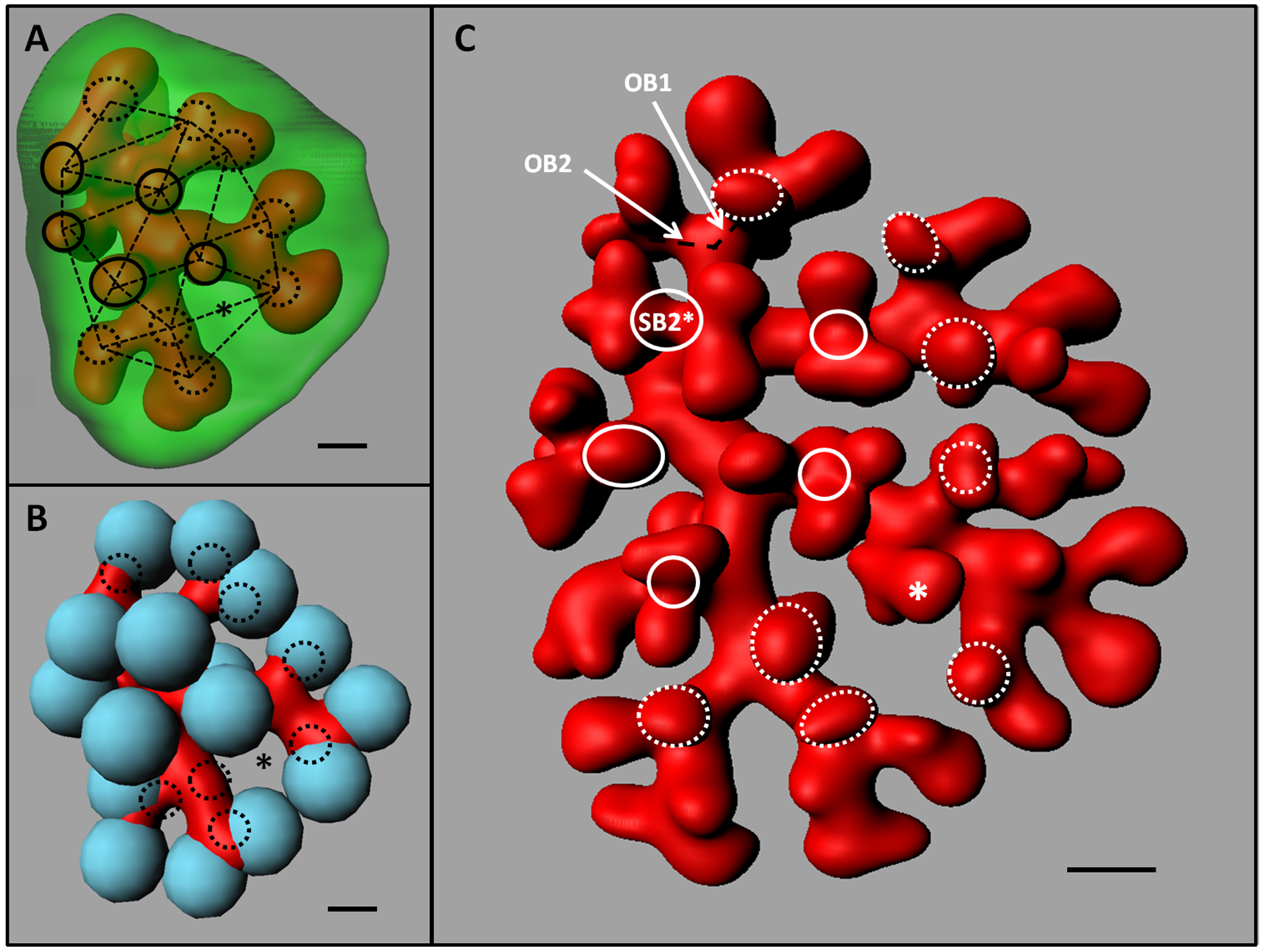

Supplement: Figure S1 — Branching sequence of the early dorsal side-branches. Panel A and B reproduce the E12.75 RCr lobe of figures 5A and 7A respectively. Panel C show a E13.25 RCr lobe. The full white circles depict branches that have already formed at E12.75 and dashed white circles show that the topography of the news buds is consistent with the E12.75 putative budding sites (dashed black circles, panel A). The gap in the meshwork (black star, panel A and B) is filled subsequently by an additional posterior lineage that gives rise quickly to a dorsal branch (white star). Panel C also show the outcome of the SB2* optional lineage. Of note, the orthogonal bifurcation forming “OB1” and “OB2” turn into planar bifurcation and give rise subsequently to ventral and dorsal branches. (TIF) [file pone.0041643.s001.tif]

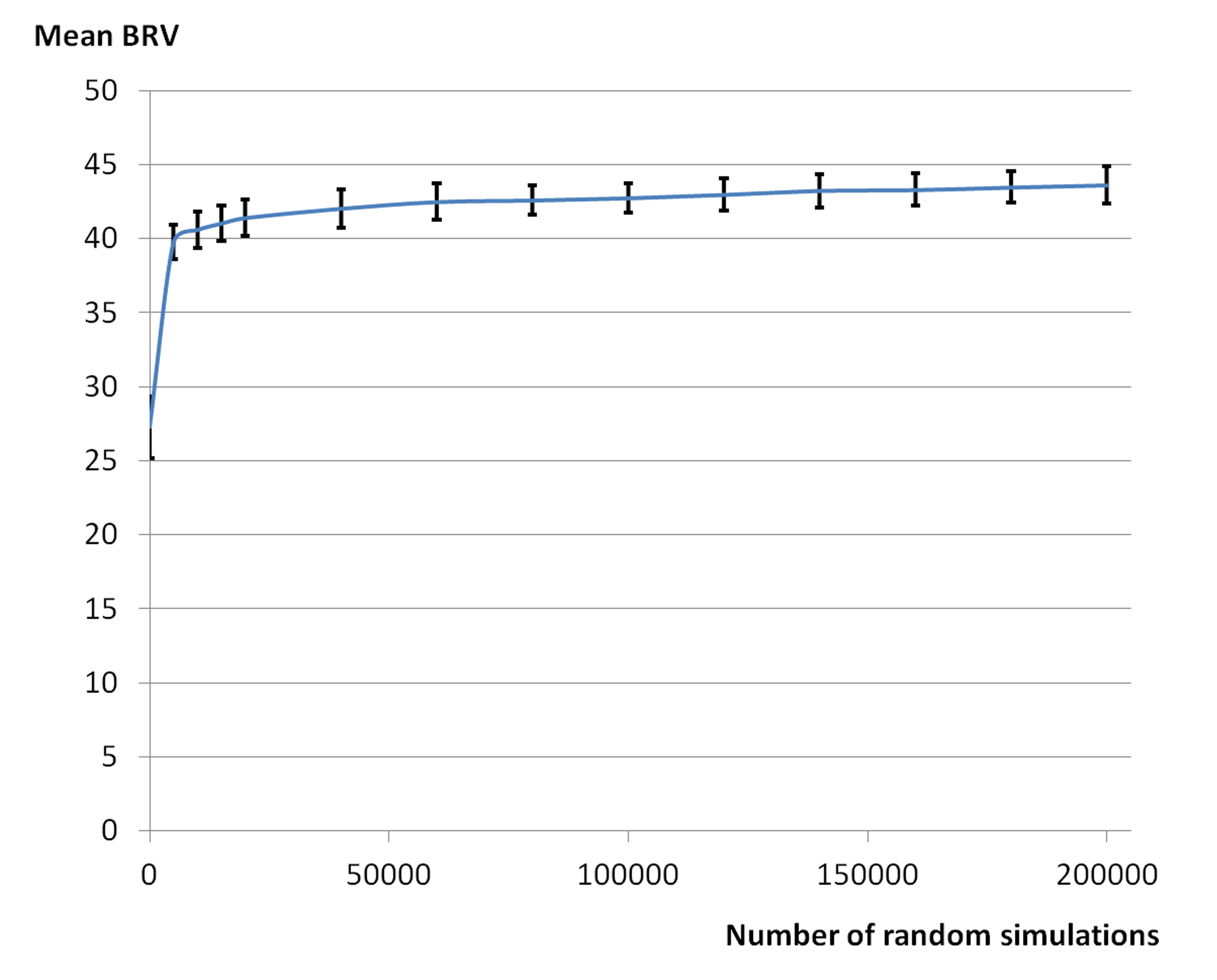

Supplement: Figure S2 — Random series thresholding. Given N points (the bud tip number) in a RCr volume V, there is a infinite number of configurations of the N positions in V. We computed X configurations at random of N points in V (here a E12.25 RCr lobe), where X was comprised between 1 and 200000. For each configuration, we calculated the space filling criterion K and then extracted the best random value (BRV) of the series. For each X value we repeated this procedure 100 times. X-axis represent the X values and Y-axis the mean BRV. Standard deviation is reported for each BRV mean. Between X = 100.000 and X = 200.000, the BRV value only increases by 1 µm, indicating that the precision gain is about 2% while the number of simulation doubles. This strongly suggests a convergence of BRV to theoretical optimal value. (TIF) [file pone.0041643.s002.tif]
